# A case-case study on sinonasal cancer prevention: effect from dust reduction in woodworking and risk of mastic/solvents in shoemaking

**DOI:** 10.1186/s12995-016-0124-7

**Published:** 2016-07-21

**Authors:** Enzo Emanuelli, Enrico Alexandre, Diego Cazzador, Vera Comiati, Tiziana Volo, Alessia Zanon, Maria Luisa Scapellato, Mariella Carrieri, Alessandro Martini, Giuseppe Mastrangelo

**Affiliations:** Department of Otolaryngology and Endoscopic Surgery of the Upper Airways, University Hospital of Padua, Via Giustiniani 2, 35100 Padua, Italy; Department of Occupational Medicine, University Hospital of Padua, Via Giustiniani 2, 35100 Padua, Italy

**Keywords:** Wood dust, Wood industry, Leather dust, Mastic solvent, Shoe industry, Sinonasal cancer, Occupational risk, Case-case study

## Abstract

**Background and aims:**

Sinonasal cancers (SNCs) are rare neoplasms, accounting for about 3 % of head and neck cancers, with squamous cell carcinoma (SCC) and adenocarcinoma (ADC) as the most common subtypes. ADCs present strong associations with occupational wood dust exposure. Preventive measures have progressively reduced wood dust concentrations in workplaces but no study has evaluated the effectiveness of such interventions. Few studies indicate associations between ADC and exposure to solvents, which is common in the shoe industry, but this hypothesis still needs confirmation.

**Methods:**

In a case-case study, we contrasted 32 ADCs against 21 Non-Adenocarcinoma Epithelial Tumors (NAETs) – all recruited from the same clinical setting (Padua’s University Hospital; period 2004–2015) – using questionnaires and clinical records to collect information on potential predictors. Non-occupational factors were age, sex, smoking, allergy and chronic sinusitis. Occupational factors were intensity and frequency of wood dust exposure, protection from wood dust, type of wood (in woodworking); frequency of exposure to leather dust or mastic/solvent (in shoemaking). Odds-ratio (OR), 95 % confidence interval (95 % CI) and two-tail p-values were obtained through stepwise backward logistic regression for each industry, always using as reference patients never employed in either trade and adjusting for non-occupational risk factors.

**Results:**

Adjusted OR was 22.5 (95 % CI = 3.50–144; *p* = 0.001) and 9.37 (95 % CI = 1.29–67.6; *p* = 0.026), respectively, in patients with low or high degree of protection against wood dust. In the shoe industry, adjusted OR was 1 and 18.8 (95 % CI = 1.29–174; *p* = 0.030), respectively, in patients with low or high exposure to only mastic/solvent; and 1 and 22.5 (95 % CI = 2.07–244; *p* = 0.011), respectively, in patients with low or high exposure to only leather dust.

**Discussion and conclusions:**

The questionnaire used was able to estimate with simple algorithms past exposures in wood and footwear industries. The case-case design considerably increased the validity of this small study. Results in this study were always consistent with the extant literature; this could support reliability of novel findings. In woodworking, respiratory protective equipment and local exhaust ventilation reduced the risk of occupational SNC; in footwear manufacture, where preventive interventions were seldom adopted, SNC risk was significantly greater for high exposure from mastic/solvent and leather dust.

**Electronic supplementary material:**

The online version of this article (doi:10.1186/s12995-016-0124-7) contains supplementary material, which is available to authorized users.

## Background

Sinonasal cancers (SNCs) are rare neoplasms that account for about 3 % of all cancers of the head and neck. The majority (59 %) of SNCs diagnosed in the USA between 2004 and 2008 were epithelial neoplasms, with squamous cell carcinoma (SCC) and adenocarcinoma (ADC) being the most common subtypes, accounting for 38 % and 10 % of all sinonasal cancers, respectively [1].

SNCs have been associated with various occupational risk factors. In Monograph 100, the International Agency for Research on Cancer (IARC) classified wood dust, leather dust, nickel compounds, isopropyl alcohol production, radium 226 and 228 and their relative decay products as Group 1 carcinogens for sinonasal mucosa. The numerous epidemiological studies on occupational exposure and sinonasal cancer risk have been recently reviewed [2–4]. In a meta-analysis, exposure to wood dust as well as to leather dust showed associations with SNC, the strongest relative risk (RR) association being with ADC (RR_pooled_ = 29.43; 95 % CI = 16.46, 52.61 for wood dust; and RR_pooled_ = 35.26; 95 % CI = 20.62, 60.28 for leather dust) [4].

Preventive measures to reduce wood dust concentration have been progressively introduced. For example, in the furniture industry of Viborg County, Denmark, two cross-sectional studies investigated wood dust exposure six years apart (winter 1997/1998, study 1; and winter 2003/2004, study 2), using 2627 personal dust exposure measures carried out in 1907 persons. The geometric mean (geometric standard deviation) of inhalable wood dust concentration decreased from 0.95 mg/m^3^ (2.05) in study 1 to 0.60 mg/m^3^ (1.63) in study 2, representing a 7 % annual decrease in dust concentration [5]. Likewise, occupational exposure in the wood processing industry in Estonia improved considerably over the years 1990–2000 [6]. The better work conditions in wood industry were generally gained with effective ventilation and consistent cleaning of the work areas [5, 6].

In data from the 1989–2009 nation-wide Netherlands Cancer Registry, the incidence of both SCC and ADC decreased gradually in males, while in females the incidence of SCC increased. The incidence of adenocarcinoma remained stable for both sexes [7]. Since ADC in males is likely related to occupational exposures, the decrease could be attributed to the implementation of measures preventing exposure or to the decreasing number of workers in the furniture and leather industry or the building industry. To the best of our knowledge, no study has yet addressed this issue.

A significant association and a dose–response relationship between exposure to organic solvents and sinonasal ADC has been observed in one study [8]. This finding, which supports the hypothesis of a causal relationship between solvents and ADC, still needs confirmation.

Exposure to mastic/solvents is common in the shoe industry; both woodworking and shoe industry are well represented in the Veneto region. We therefore aimed to investigate in these industrial sectors the SNC risk resulting from the two new aspects of occupational experience: 1) use of personal protective equipment/presence of local exhaust ventilation in wood industry and 2) exposure to glues/organic solvents in shoe industry.

## Methods

### Patient selection

The source of patients was the database of the Departments of Otolaryngology and Endoscopic Surgery of Airways of Padua’s University Hospital. Out of 92 patients with SNCs observed between 2004 and 2015, 26 were excluded because of histology (mesenchymal cancers or epithelial cancers not of sinonasal mucosa, i.e. melanoma). The remaining 66 patients (affected by sinonasal ADC, SCC, undifferentiated carcinoma, mucoepidermoid carcinoma and adenoid cystic carcinoma) were invited by phone to participate in the study; 53 patients (40 males and 13 females) agreed to participate. Written informed consent was obtained before examination. The sample included eight (15 %) decedents; for dead or elderly/disable patients, a letter with the informed consent was sent to the patient’s home, together with a return addressed envelope. For dead patients, closest next of kin signed the informed consent and answered the questionnaire telephonically. Elderly/disabled patients, who were not able to be interviewed face-to-face at the hospital, completed the questionnaire telephonically.

### Study design

As already reported, ADC is largely associated with woodworking and leather working, while other Non-Adenocarcinoma Epithelial Tumors (NAETs) can be viewed as a background risk. We therefore used a case-case study design contrasting two groups of patients: 32 ADCs and 21 NAETs (the latter including SCC and a few cases of adenoid cystic carcinoma, undifferentiated carcinoma and mucoepidermoid carcinoma) for their occupational exposure. Since the two groups were recruited from the same clinical setting, they are likely to be similar in terms of geographical background, social class and symptoms that lead the patients to seek clinical attention. Moreover, since NAET and ADC share non-occupational risk factors (smoking, age, others), the two samples were well matched for such risk factors, reducing background noise and increasing both the validity and specificity of the association [9].

### Variables

The site of onset of both ADCs and NAETs and the histologic subtypes of ADCs were obtained from clinical records. The site of onset was coded as: 0 = ethmoid; 1 = maxillary; 2 = nasal septum. ADC subtypes were coded as: 0 = Intestinal-Type Adenocarcinoma (ITAC); 1 = Non Intestinal-Type Adenocarcinoma (non-ITAC).

All other variables used in the analysis were collected through interview-based questionnaires (Additional file 1). Non-occupational risk factors were:

- age at diagnosis (continuous variable) and gender (1 = male; 0 = female);
- clinical history for allergies, chronic polypoid sinusitis and chronic non-polypoid sinusitis (1 = present; 0 = absent);
- smoking: pack-years (= cigarettes per day/20 × years of smoking) and a variable that was 0 for never smokers; 0.5 for ex-smokers quitting >15 years from interview; 1 for current smokers and former smokers quitting < 15 years.

Using the same questionnaire, we collected the following circumstances of past occupational exposures.

Wood industry

- Intensity. We considered calendar year of first exposure as a surrogate of intensity of wood dust exposure because, depending on technological advancements, it has been reduced over time. Four classes were coded: 0 (subjects never exposed to wood or leather dusts); 1 (year of first exposure ≥1971); 2 (1956 to 1970); 3 (≤1955).
- Frequency. We presumed that the jobs involving high exposure to wood dust were cutting, sanding, planing and use of compressed air; each job was scored as a dichotomous variable (0 = No; 1 = Yes). Then, we collected the workshift frequency (hours per shift/8) of each high exposure job. Finally, we multiplied (job × frequency) summing up as many products as were necessary to take into account the overall work experience of each patient. The latter index ranged from 0 to 1. The frequency of high exposure was then coded: 0 (for subjects never exposed to wood dusts nor leather dusts); 1 (frequency ≤0.5); 2 (frequency >0.5).
- Protection. The original variables were: frequency of high exposure (as above); personal protective equipment (PPE) such as masks (codes = 0/1); and presence of local exhaust ventilation (LEV) (codes = 0/1). The level of protection against wood dust was calculated as: ((PPE + (2 × LEV)) × frequency), summing up all the exposed tasks. The index ranged from 0 to 3.75, increasing with increasing level of protection. The variable was recoded as: 0 (subjects never exposed to wood dusts nor leather dusts); 1 (≥2.6, most protected subjects); 2 (0.1–2.5, least protected subjects).
- Wood type. Coded as 0 for subjects never exposed to wood nor leather dusts; 1 (softwood or mixed wood types); 2 (only hardwoods).
- Duration. Coded as 0 for subjects never exposed to wood nor to leather dusts; 1 and 2 for those with <15 and ≥15 years of exposure to wood dust, using as threshold the value suggested by Binazzi et al. [4].

Shoe industry

- Frequency (leather dust). The jobs presuming to involve a high exposure to leather dust (cutting, polishing and sole preparation) were converted in 0/1 variables and multiplied by the corresponding frequencies (hours per workshift/8). The products were summed up into an index ranging from 0.05 to 1. Three classes of increasing exposure were coded: 0 (subjects never employed into wood industry nor leather industry); 1 (frequency ≤0.05, least exposed workers); 2 (frequency >0.05, most exposed workers).
- Frequency (mastic solvents). The jobs presuming to involve a high exposure to mastic and/or solvents (shoe-assembling and gluing) were converted in 0/1 variables that were multiplied with the corresponding frequency (hours per workshift/8). The products were summed up into an index ranging from 0.05 to 1. Three classes of increasing exposure were coded: 0 (subjects never employed into wood industry nor leather industry); 1 (frequency ≤0.5, least exposed workers); 2 (frequency >0.5, most exposed workers).

### Statistical analysis

Analyses were performed within each specific industrial sector (woodworking or shoe industry). The reference group for all analyses consisted of patients never exposed to wood and leather dust. Seven logistic regression models were built in which the dependent variable was dichotomous (1 = ADC; 0 = NAET) and the primary variables were: exposure (model 1); frequency (model 2); protection (model 3); type of wood (model 4); and duration of exposure in wood industry (model 5); frequency of leather dust exposure in shoe industry (model 6); and frequency of mastic solvent exposure in shoe industry (model 7). Some employees less exposed to mastic solvent were mainly involved in tasks that had higher exposures to leather dust, and vice versa. Therefore, the analysis according to models 6 and 7 were repeated excluding those with simultaneous high exposure to the other factor. The non-occupational risk factors (smoking, gender, age at diagnosis, allergies, chronic sinusitis) entered every model. Backward stepwise selection was used to exclude non-statistically significant variables and determine the best-fit model. The statistical procedure provided the odds-ratio (OR) together with 95 % confidence interval (95 % CI) and two-tail p-value. Statistical significance was set at *p* < 0.05; all analyses were performed with STATA 13.0 software.

## Results

Table 1 shows the main characteristics of ADC and NAET patients. It can be seen that industrial sector was highly related with the development of ADC, while all non-occupational risk factors were not. The site of onset for ADCs was the ethmoid sinus (100 %); for NAETs the most frequent site was the maxillary sinuses (38 %), followed by nasal septum (33 %), and ethmoid (29 %). ADC cases were mostly ITAC (94 %).

**Table 1:** Main characteristics of patients with adenocarcinoma (ADC), non-adenocarcinoma epithelial tumor (NAET) and in the whole sample

*Patients’ characteristics*		ADC	NAET	Whole sample	Chi square (*p*-value)
		Number (%)	Number (%)	Number (%)	
*Industrial sector*	*Wood*	16 (50)	5 (24)	21 (40)	14.60 (0.001)
	*Leather*	11 (34)	2 (10)	13 (25)	
	*Other*	5 (16)	14 (67)	19 (36)	
*Age (years)*	*≤59*	8 (25)	10 (48)	18 (34)	4.17 (0.125)
	*60–74*	8 (25)	6 (29)	14 (26)	
	>74	16 (50)	5 (23)	21 (40)	
*Smoking (pack-years)*	*0*	17 (53)	14 (67)	31 (58)	2.67 (0.263)
	*≤18*	6 (19)	5 (24)	11 (21)	
	*>18*	9 (29)	2 (9)	11 (21)	
*Sex*	*M*	26 (81)	14 (67)	40 (75)	1.46 (0.227)
	*W*	6 (19)	7 (33)	13 (25)	
*Chronic Sinusitis*	*Yes*	6 (19)	4 (19)	10 (19)	0.001 (0.978)
	*No*	26 (81)	17 (81)	43 (81)	
*Allergies*	*Yes*	4 (13)	2 (10)	6 (11)	0.11 (0.738)
	*No*	28 (87)	19 (90)	47 (89)	
*Site of onset*	*Ethmoid*	32 (100)	6 (29)	38 (72)	
	*Maxillary*	0	8 (38)	8 (15)	
	*Septum*	0	7 (33)	7 (13)	
*ADC subtype*	*ITAC* ^*a*^	30 (94)	-	-	
	*Non-ITAC*	2 (6)	-	-	

^*a*^Intestinal-Type Adenocarcinoma

Table 2 shows the distribution of patients (ADC or NAET) as well as the OR with 95 % CI and p-value of developing ADC in wood industry. In model 1, the highest ORs were observed for the remotest years of first exposure to wood dust. In model 2, ORs increased with increasing frequency of high exposure to wood dust. In model 3, ORs tended to decrease with increasing level of protection from wood dust. In model 4, exposure to hardwood and (to a lesser extent) softwood significantly increased the risk of ADC. In model 5, ORs tended to increase with increasing duration of exposure to wood dust (OR being 1 in the subgroup with <15 years of exposure because there were no ADC cases). Non-occupational risk factors did not enter in any model.

**Table 2:** Distribution of patients and results of five models of logistic regressions (wood industry only)

Terms	ADC	NAET	OR	95 % CI	*p*-value
*Model 1. First exposure to wood dust*					
*Reference*	4	15	1	-	-
*1970-today*	5	2	9.37	1.29–67.6	0.026
*1956–1970*	6	1	22.5	2.06–245.	0.011
*Pre-1956*	6	1	22.5	2.06–245.	0.011
*Model 2. Frequency of exposure to wood dust*					
*Reference*	4	15	1	-	-
*Low*	7	2	13.1	1.92–89.5	0.009
*High*	10	2	18.8	2.87–122.	0.002
*Model 3. Protection from wood dust*					
*Reference*	4	15	1	-	-
*Low*	12	2	22.5	3.50–144.	0.001
*High*	5	2	9.37	1.29–67.6	0.026
*Model 4. Wood used*					
*Reference*	4	15	1	-	-
*Other woods*	8	3	10.0	1.78–56.1	0.009
*Hardwood*	9	1	33.8	3.22–351.	0.003
*Model 5. Duration of exposure to wood dust*					
*Reference*	4	15	1	-	-
*<15 years*	4	0	1.0	-	.
*≥15 years*	13	4	12.2	2.52–58.7	0.002

*ADC* adenocarcinoma, *NAET* non-adenocarcinoma epithelial tumor, *OR* odds-ratio, 95%CI 95 % confidence interval

Table 3 shows the distribution of patients (ADC or NAET) as well as the OR with 95%CI and p-value of developing ADC in the shoe industry. ORs were found to increase with increasing frequency of estimated exposure to both leather dust (model 6) and mastic/solvent (model 7) although, in the latter model, the relationship was not monotonic probably because employees lesser exposed to mastic solvent had higher exposure to leather dust. Non-occupational risk factors did not enter any of these models.

**Table 3:** Distribution of patients and results of two models of logistic regressions (shoe industry only)

Terms	ADC	NAET	OR	95 % CI	*p*-value
*Model 6. Frequency of exposure to leather dust*					
*Reference*	4	15	1	-	-
*Low*	5	1	18.75	1.67–209.	0.017
*High*	6	1	22.5	2.06–244.	0.011
*Model 6. Frequency of exposure to leather dust (no exposure to mastic/solvent)*					
*Reference*	4	15	1	-	-
*Low*	1	0	1	-	-
*High*	6	1	22.5	2.06–244.	0.011
*Model 7. Frequency of exposure to mastic/solvent*					
*Reference*	4	15	1	-	-
*Low*	7	1	26.25	2.45–280.	0.007
*High*	4	1	15.00	1.29–174.	0.030
*Model 7. Exposure to mastic/solvent (no exposure to leather dust)*					
*Reference*	4	15	1	-	-
*Low*	1	0	1	-	-
*High*	4	1	15.00	1.29–174.	0.030

*ADC* adenocarcinoma, *NAET* non-adenocarcinoma epithelial tumor, *OR* odds-ratio, 95%CI 95 % confidence interval

## Discussion and Conclusions

We contrasted two groups of patients (ADC against NAET) for circumstances of exposure in wood and shoe industry. The role of traditional risk factors was always confirmed; regarding the two new hypotheses, we found that use of PPE and/or presence of LEV reduced the risk of occupational SNC in wood industry, while exposure to mastic/solvents increased such risk in the shoe industry.

### Internal validity

A weakness of the present study is the limited size. However, according to Curtis [9], the choice of a case-case study rather than the classic case-control study could lower the background noise because two similar diseases share most of non-occupational risk factors. In fact, as shown in Table 1, there were no differences between ADC and NAET groups for gender, age at diagnosis, clinical history of allergies, chronic polypoid or non-polypoid sinusitis, and smoking habits. Thus, despite the low number of our subjects, the case-case design has considerably increased the validity of the study, overcoming the several difficulties that often affect investigations on these rare diseases.

The proportion of NAET patients exposed to risks was about 20 % in our study group in contrast to 8 % in a clinical series reported by Bonzini et al. [10]. Since the corresponding percentage was about 2.2 % in the general population of the Veneto region in 1996 (about 100.000 workers in 4.500.000 inhabitants); using NAETs as references probably has underestimated the true risk of developing ADC.

Besides SCC, we included in the reference group cases with different histologies (adenoid cystic carcinoma, undifferentiated carcinoma and mucoepidermoid carcinoma). Although this could represent a major limit to the study, given the low number of patients and the scarce evidence of association between these histologies and exposure to wood and leather dust [10], it is unlikely that such inclusion could have severely biased our results.

A questionnaire was specifically designed for this study. In agreement with Hernberg [11], we assumed that calendar year of first exposure could be a reliable surrogate of intensity of exposure, because occupational hygiene in general has improved in many countries and work methods have changed substantially over the years. In agreement with Schluenssen [5], we considered sanding, cutting, sanding and cutting, mixed task, and use of compressed air work as tasks involving high level of exposure to wood dust – moreover we also collected the frequency (hours per workshift/8) spent in each high exposure task. In the same study [5], manual assembling/packing, sanding with adequate exhaust ventilation, adequate exhaust ventilation, vacuum cleaning of machines and special cleaning staff were found to decrease wood dust concentration. Accordingly, we built an algorithm to characterize protection against wood dust exposure, using the presence of LEV and use of PPE. Building an algorithm of protection in shoe sector was not feasible because PPE and respiratory protective equipment were seldom used by our subjects and in literature [12]. In short, using literature data we created a questionnaire that was able to estimate with relatively simple algorithms past exposures to occupational pollutants in wood and leather industry.

Collecting occupational history from next-of-kin should could lower the quality of information because relatives tend to over- or under-estimate such exposure [11]. The latter fact increases the non-differential misclassification that underestimates the true OR. Despite this fact, our findings resulted statistically significant.

### Interpretation of findings

As clearly reported in Table 1, ITAC arisen from ethmoid sinus was the commonest subtype of ADC in our study, in agreement with extant literature [10, 13–15]. Notwithstanding, SNCs that originate from other sinonasal sites can be associated with occupational exposure [4, 16]. For woodworking, moreover, we were able to confirm the increasing trend of SNC with duration of exposure to wood dust (model 5) [4]. Our results were coherent with extant literature [2–4], and this could support the reliability of the new findings reported in this study in spite of the low number of cases and the wide confidence intervals of risk estimates.

The novel finding was the evidence that PPE and/or LEV could be effective in preventing occupational SNCs. It could be noted, however, that OR was still as high as 9 in the group with maximal protection (model 3). This finding, which also suggests that further improvements need to be done, could be the residual effect of high wood dust exposures prior to 1981 − year in which woodworking was first classified by IARC as class I carcinogen − that became manifest after the typical long latency of these tumors. Likely, in fact, woodworking industries started providing their employees with PPEs or installing exhaust systems or more technically advanced protective machines only starting in 1981. In our subjects, moreover, when intensity or duration of exposure, frequency of exposure and protection from exposure were forced with the non-occupational risk factors in a same model of stepwise logistic regression, protection was the only significant predictor (same results of model 3). Therefore, the ultimate risk for sinonasal cancer in woodworking was the lack of primary prevention. A review of the literature shows no other reports on this topic. In Italy, in the sector of wood and cabinet making, the number of employees increased progressively from the year 1951 to 1981 to then decrease to levels of 1961 in the year 2001 (seriestoriche.istat.it). The corresponding values are not available for the Veneto region. Presuming that values were similar in the Veneto region and given the long latency period of the disease, the decreasing number of employees is too recent to explain the decrease of SNC risk.

In the shoe industry, the novel finding was the association between exposure to mastic/solvent and risk of developing ADC. An overestimation of such risk was unlikely because neither patients nor interviewers had any prior knowledge of organic solvents as a suspected occupational risk factor for SNC. There are in fact few reports on this issue. In a case-control study carried out in Piedmont (Italy) in 113 incident cases and 336 hospital controls, d’Errico [8] reported a significant association and a dose–response relationship between occupational exposure to organic solvents and sinonasal ADC, after controlling for age, sex, smoking and co-exposure to wood dust. Exposure to solvents was evaluated based on the information provided in the questionnaires in conjunction with two job-exposure matrices by economic sector and job title [8]. In a population-based case-control study undertaken in Southern Germany, involving 427 cases (cancers of the paranasal sinuses, cancers of the nasal cavity, nasopharyngeal cancer) and 2401 controls, Greiser [17] found a significant excess of nasal cancer in smokers after exposure to organic solvents but not a dose-response relationship. Exposure to organic solvents was determined at the time of the interview with questions about exposure during any occupation as well as by a detailed questionnaire module about specific tasks in metalwork [17]. The majority of our subjects reported exposure to solvents in absence of personal protective equipment, task barriers and mechanical ventilation. Likewise, in a study conducted in Hebron City, 81 % of the shoe factory workers had never used respiratory protective equipment and 92 % had never used work clothes; while 97 % of the workers in the shoe workshops had never used respiratory protective equipment, 94 % had never worn gloves, and 90 % had never used work clothes [12]. Other studies found that workers in footwear manufacture are routinely exposed to complex mixtures of solvents, including acetone, n-hexane, methylethylketone, and large amounts of toluene [18, 19]. None of these solvents is considered genotoxic or carcinogenic [20–22]. The results observed in this study could possibly be explained by the presence of some elastomer in adhesives such as chloroprene, which is used mainly in the footwear industry and is ranked as 2B carcinogen by IARC [23].

The duration of exposure in this industry was not analyzed because of the few cases with specific exposure to either of the substances studied. However, in a recent meta-analysis, no relationship was reported between duration of exposure and SNC risk in the shoe industry [4].

## Additional file

Additional file 1: Questionnaire of etiological evaluation for tuns. (DOCX 94 kb)

## Abbreviations

ADC: adenocarcinoma
LEV: local exhaust ventilation
NAET: non-adenocarcinoma epithelial tumor
PPE: personal protective equipment
SCC: squamous cell carcinoma; SNC, sinonasal cancer

## References

[1] Youlden DR, Cramb SM, Peters S, Porceddu SV, Møller H, Fritschi L (2013). International comparisons of the incidence and mortality of sinonasal cancer. Cancer Epidemiol.

[2] Husgafvel-Pursiainen K, Carton M, Luce D, Wolff CHJ, Holmila R, Schlünssen V et al. Sinonasal cancers. Occupational Cancers. 2014. 139-168. http://link.springer.com/chapter/10.1007%2F978-1-4471-2825-0_7.

[3] Alonso-Sardón M, Chamorro AJ, Hernández-García I, Iglesias-de-Sena H, Martín-Rodero H, Herrera C (2015). Association between occupational exposure to wood dust and cancer: a systematic review and meta-analysis. PLoS One.

[4] Binazzi A, Ferrante P, Marinaccio A (2015). Occupational exposure and sinonasal cancer: a systematic review and meta-analysis. BMC Cancer.

[5] Schlünssen V, Jacobsen G, Erlandsen M, Mikkelsen AB, Schaumburg I, Sigsgaard T (2008). Determinants of wood dust exposure in the Danish furniture industry—results from two cross-sectional studies 6 years apart. Ann Occup Hyg.

[6] Traumann A, Reinhold K, Tint P (2013). The model for assessment of health risks of dust connected with wood manufacturing in Estonia. Agron Res.

[7] Kuijpens J, Hans LP, Louwman MWJ, Peters R, Janssens GORJ, Burdorf AL (2012). Trends in sinonasal cancer in The Netherlands: More squamous cell cancer, less adenocarcinoma: A population-based study 1973–2009. Eur J Cancer.

[8] d’Errico A, Pasian S, Baratti A (2009). A case-control study on occupational risk factors for sino-nasal cancer. Occup Environ Med.

[9] Curtis D, Vine AE, McQuillin A, Bass NJ, Pereira A, Kandaswamy R (2011). Case-case genome wide association analysis reveals markers differentially associated with schizophrenia and bipolar disorder and implicates calcium channel genes. Psychiatr Genet.

[10] Bonzini M, Battaglia P, Parassoni D, Casa M, Facchinetti N, Turri-Zanoni M, et al. Prevalence of occupational hazards in patients with different types of epithelial sinonasal cancers. Rhinology. 2013;51(1):31–6. https://www.researchgate.net/profile/Mario_Turri_Zanoni/publication/235730863_Prevalence_of_occupational_hazards_in_patients_with_different_types_of_epithelial_sinonasal_cancers/links/00b7d526d35d5a908d000000. 10.4193/Rhino11.228 23441309

[11] Hernberg S. Introduction to occupational epidemiology. CRC Press; 1992.

[12] Nijem K, Kristensen P, Thorud S, Al-Khatib A, Takrori F, Bjertness E (2001). Solvent exposures at shoe factories and workshops in Hebron City, West Bank. Int J Occup Environ Health.

[13] Bimbi G, Saraceno MS, Riccio S, Gatta G, Licitra L, Cantù G (2004). Adenocarcinoma of ethmoid sinus: an occupational disease. Acta Otorhinolaryngol Ital.

[14] Khademi B, Moradi A, Hoseini S, Mohammadianpanah M (2009). Malingant neoplasms of the sinonasal tract: report of 71 patients and literature review and analysis. Oral Max Fac Surgery.

[15] Bossi P, Saba NF, Vermorken JB, Strojan P, Pala L, de Bree R, Takes RP (2015). The role of systemic therapy in the management of sinonasal cancer: A critical review. Cancer Treat Rev.

[16] Mensi C, Consonni D, Sieno C, De Matteis S, Riboldi L, Bertazzi PA (2013). Sinonasal cancer and occupational exposure in a population-based registry. Int J Otolaryngology.

[17] Greiser EM, Greiser KH, Ahrens W, Hagen R, Lazszig R, Maier H (2012). Risk factors for nasal malignancies in German men: the South-German Nasal cancer study. BMC Cancer.

[18] Uuksulainen SO, Heikkilä PR, Olkinuora PS, Kiilunen M (2002). Self-reported occupational health hazards and measured exposures to airborne impurities and noise in shoe repair work. Int J Occup Environ Health.

[19] Pitarque M, Vaglenov A, Nosko M, Hirvonen A, Norppa H, Creus A (1999). Evaluation of DNA damage by Comet assay in shoe-workers exposed to toluene and other organic solvents. Mutat Res.

[20] ASTDR (Agency for Toxic Substances and Disease Registry)-ToxFAQs, Toxicological Profile for: 2-butanone (1995). Nhexane (1999). Toluene (2001). Acetone (2002).http://www.atsdr.cdc.gov/substances/index.asp. Accessed 19 Jan 2016

[21] WHO (World Health Organization). Air quality Guidelines, second edition, Chapter 5.14. Toluene. Regional Office for Europe, Copenhagen, Denmark. http://www.who.int/. 2000. Accessed 19 Jan 2016

[22] EPA (Environmental Protection Agency). In Support of Summary Information on the Integrated Risk Information (IRIS). Toxicological Review ofMethyl Ethyl Ketone. http://epa.gov/iris. 2003. Accessed 16 Jan 2016

[23] International Agency for Research on Cancer (IARC). IARC Monographs on the evaluation of carcinogenic risks to humans. Re-evaluation of some organic chemicals, hydrazine and hydrogen peroxide. Lyon: IARC, 71. 1999. PMC7681305 10507919

