# Supplementary material for: A case-case study on sinonasal cancer prevention: effect from dust reduction in woodworking and risk of mastic/solvents in shoemaking
Source: J Occup Med Toxicol. 2016 Jul 21;11:35. doi: 10.1186/s12995-016-0124-7 (PMC4957368; doi:10.1186/s12995-016-0124-7)
Supplement: Additional file 1: — Questionnaire of etiological evaluation for tuns. (DOCX 94 kb) [file 12995_2016_124_MOESM1_ESM.docx]

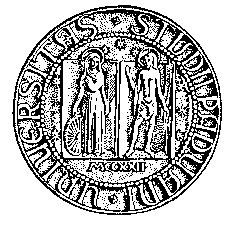


AZIENDA OSPEDALIERA DI PADOVA

UNIVERSITY OF PADUA

# *QUESTIONNAIRE OF ETIOLOGICAL EVALUATION FOR TuNS*

Patient Code.............................

Name and Surname...........................................................................................

Place of birth ............................................................ Date of birth......../ ......../ ..............

Sex: M □ F□

Last address: Street ........................................ City...............................………

Phone number ............................................ Mobile Phone…………………………………..

General practitioner ……………………………………………………………………………….

Address ………………………………………………………… Phone number ……………………

Date of interview ......../ ......../............Place of interview................................................................

Interviewer............................................................... Institution .........................................................

Role (qualification)…………………………………………….

About the person who answers (if different from the patient)

Name and Surname...........................................................................................

Sex M □ F □ Degree of kinship ............................................................

Address .............................................................................. Phone number. ……………………….

If the interview did not take place:

□ Untraceable

□ Refusal to partecipate

□ Psychophysical inability

□ Deceased, relatives not found

□ Deceased, relatives refused to partecipate

**Clinical and sinonasal history**

positive rhino-sinusal history?

Yes□ No□

If Yes:

Chronic not polypoid rhinosinusitis □

Chronic polypoid rhinosinusitis □

Turbinate hypertrophy □

Clinical diagnosis (cancer site):

Histological diagnosis: Date of histological diagnosis:

Stadiation:

Surgical treatment (to be specified):

Radiotherapy: Yes □ No □ If yes, specify

chemotherapy: Yes □ No □ If yes, specify

Recurrence: Yes □ No □ If yes, specify

Date of recurrence:

Other prognostic indicators

AJCC stadiation(7^th^ ed. 2010) I □ II □ III □ IVa □ IVb □ IVc □

Grading G1 □ G2 □ G3 □

Histological subtype (if ADC) colonic □ mucinous □ papillary □ solid □ mixed □ non-ITAC □

Skull-base/orbit spread Yes □ No □

Margins + Yes □ No □

Local recurrence after first treatment Yes □ No □

(other site of relapse) if yes, which treatment_________________________

if yes, disease-free interval _____________________

**Non occupational risk factors**

Smoking habit

Current smoker Yes □ No □

Smoking Period from …… to ………. cigarettes □ n° cigarettes /day …….

Smoking Period from …… to ………. cigarettes □ n° cigarettes /day …….

Former smoker □ for years (n°) ………………..

**Occupational history – general summary**

Currently working? Yes □ No □ if not, specify the reason:.....................

Attach copy of employee's official work history, if available.

**Hazardous occupation**

Have you ever worked in:

| 1 | Wood and cabinet (cabinet, sawmill, chairs production, wooden boats) | Yes □ No □ Occasional □ |
| --- | --- | --- |
| 2 | Shoe and leather industry | Yes □ No □ Occasional □ |
| 3 | Tannery industry | Yes □ No □ Occasional □ |
| 4 | Metallurgic industry (nickel refining, production stainless steels, alloys and superalloys, production iron-chromium alloys and high chromium content alloys) | Yes □ No □ Occasional □ |
| 5 | Metal processing industry (welding, galvanic processing, machining) | Yes □ No □ Occasional □ |
| 6 | Chemical industry (in particular, production and usage of  chromates) | Yes □ No □ Occasional □ |
| 7 | Textile industry (carding, spinning / weaving) | Yes □ No □ Occasional □ |
| 8 | Forestry | Yes □ No □ Occasional □ |
| 9 | Other (describe) | Yes □ No □ Occasional □ |

Does the National Institute for Insurance against Accidents at Work (INAIL) has ever recognized any occupational disease of yours? Yes □ No □

If yes, which one? …………………………………………………………………………………………...

…………………………………………………………………………………………………………

**Job-specific schedule**

1. WOOD AND CABINET

| Company* | | | Start | | End |
| --- | --- | --- | --- | --- | --- |
| Job sector/  Type of production |  | | | | |
| Sanding operation | Sanding with machines □ Hand sanding □ | | | | |
|  | N° hours/day how many years <5 □ 5-10 □ 10-20 □ 20-30□ >30 □ | | | | |
|  | Presence of exhaust systems? Yes localized □ Yes generalized □ No □ | | | | |
|  | Usage of dust masks: Frequent w/ filter□ Frequent w/o filter□ No/rare□ | | | | |
| Cutting operation *(to be specified)* | N° hours/day how many years <5 □ 5-10 □ 10-20 □ 20-30□ >30 □ | | | | |
|  | Presence of exhaust systems? Yes localized □ Yes generalized □ No □ | | | | |
|  | Usage of dust masks: Frequent w/ filter□ Frequent w/o filter□ No/rare□ | | | | |
| other production *(to be specified)* | N° hours/day how many years <5 □ 5-10 □ 10-20 □ 20-30□ >30 □ | | | | |
|  | Presence of exhaust systems? Yes localized □ Yes generalized □ No □ | | | | |
|  | Usage of dust masks: Frequent w/ filter□ Frequent w/o filter□ No/rare□ | | | | |
| other production *(to be specified)* | N° hours/day how many years <5 □ 5-10 □ 10-20 □ 20-30□ >30 □ | | | | |
|  | Presence of exhaust systems? Yes localized □ Yes generalized □ No □ | | | | |
|  | Usage of dust masks: Frequent w/ filter□ Frequent w/o filter□ No/rare□ | | | | |
| Utilized machinery | band saws□ circular saws□ toupies □ sanders □  milling machines □ buffing machines □ other*(specify)* □ | | | | |
| Cleaning of workplace/ machineries | Usage of compressed air: n° of hours/day years<10 □ 10-20 □ 20-30 □ >30 □ | | | | |
|  | Usage of vacuum cleaners: n° of hours/day years? <10□ 10-20□ 20-30 □ >30□ | | | | |
|  | Usage of dust masks: Frequent w/ filter □ Frequent w/o filter□ No/rare□ | | | | |
| Utilized woods | softwoods | hardwoods | | exoticwoods | |
|  | Yes □ Which one ( % of usage)? | Yes □ Which one ( % of usage)? | | Yes □ Which one ( % of usage)? | |
|  | Usage of particle board ?Yes □ No □ | | | | |
| Painting procedures | If yes, which % of the daily working time? | | | | |
|  | polyester paints □ polyurethanic paints □ nitro paints □ other (specify)□ | | | | |
| Exposure to any other chemical compounds? *(in particular to solvents)* Yes □ No □  *(to be specified)* | | | | | |

*Compile a job-specific schedule for every company of this sector given in full professional history

**Job-specific schedule**

1. FOOTWEAR INDUSTRY(LEATHER)

| Company* | | Start | End |
| --- | --- | --- | --- |
| Job sector/  Type of production |  | | |
| Cutting operations | N° hours/day how many years <5 □ 5-10 □ 10-20 □ 20-30□ >30 □ | | |
|  | Presence of exhaust systems? Yes localized □ Yes generalized □ No □ | | |
|  | Usage of dust masks: Frequent w/ filter□ Frequent w/o filter□ No/rare□ | | |
| Folding and binding operations | N° hours/day how many years <5 □ 5-10 □ 10-20 □ 20-30□ >30 □ | | |
|  | Presence of exhaust systems? Yes localized □ Yes generalized □ No □ | | |
|  | Usage of dust masks: Frequent w/ filter□ Frequent w/o filter□ No/rare□ | | |
| Preparation of soles or heels | N° hours/day how many years <5 □ 5-10 □ 10-20 □ 20-30□ >30 □ | | |
|  | Presence of exhaust systems? Yes localized □ Yes generalized □ No □ | | |
|  | Usage of dust masks: Frequent w/ filter□ Frequent w/o filter□ No/rare□ | | |
|  | Usage of glues? Yes (water glues) □ Yes (solvent glues)□ No □ | | |
| Assembly | N° hours/day how many years <5 □ 5-10 □ 10-20 □ 20-30□ >30 □ | | |
|  | Presence of exhaust systems? Yes localized □ Yes generalized □ No □ | | |
|  | Usage of dust masks: Frequent w/ filter□ Frequent w/o filter□ No/rare□ | | |
|  | Usage of glues? Yes (water glues) □ Yes (solvent glues)□ No □ | | |
| Other production *(to be specified)* | N° hours/day how many years <5 □ 5-10 □ 10-20 □ 20-30□ >30 □ | | |
|  | Presence of exhaust systems? Yes localized □ Yes generalized □ No □ | | |
|  | Usage of dust masks: Frequent w/ filter□ Frequent w/o filter□ No/rare□ | | |
|  | Usage of glues? Yes (water glues) □ Yes (solvent glues)□ No □ | | |
| Cleaning of workplace/ machineries | Usage of compressed air: n° of hours/day years<10 □ 10-20 □ 20-30 □ >30 □ | | |
|  | Usage of vacuum cleaners: n° of hours/day years? <10□ 10-20□ 20-30 □ >30□ | | |
|  | Usage of dust masks: Frequent w/ filter□ Frequent w/o filter□ No/rare□ | | |
| Exposure to any other chemical compounds? *(in particular to solvents)* Yes □ No □  *(specify)* | | | |

* Compile a job-specific card for every company of this sector given in full professional history

**Job-specific schedule**

1. TANNERY INDUSTRY

| Company* | | Start | End |
| --- | --- | --- | --- |
| Job sector/  Type of production |  | | |
| Drum tanning operations | N° hours/day how many years <5 □ 5-10 □ 10-20 □ 20-30□ >30 □ | | |
|  | Presence of exhaust systems? Yes localized □ Yes generalized □ No □ | | |
|  | Usage of dust masks: Frequent w/ filter□ Frequent w/o filter□ No/rare□ | | |
|  | Exposed to: Tannins □ Chromium tanning compounds □ other (see below) □ | | |
| Mechanical processing (fleshing, smashing or screeding) | N° hours/day how many years <5 □ 5-10 □ 10-20 □ 20-30□ >30 □ | | |
|  | Presence of exhaust systems? Yes localized □ Yes generalized □ No □ | | |
|  | Usage of dust masks: Frequent w/ filter□ Frequent w/o filter□ No/rare□ | | |
| Other production *(to be specified)* | N° hours/day how many years <5 □ 5-10 □ 10-20 □ 20-30□ >30 □ | | |
|  | Presence of exhaust systems? Yes localized □ Yes generalized □ No □ | | |
|  | Usage of dust masks: Frequent w/ filter□ Frequent w/o filter□ No/rare□ | | |
| Other production *(to be specified)* | N° hours/day how many years <5 □ 5-10 □ 10-20 □ 20-30□ >30 □ | | |
|  | Presence of exhaust systems? Yes localized □ Yes generalized □ No □ | | |
|  | Usage of dust masks: Frequent w/ filter□ Frequent w/o filter□ No/rare□ | | |
| Cleaning of workplace/ machineries | Usage of compressed air: n° of hours/day years<10 □ 10-20 □ 20-30 □ >30 □ | | |
|  | Usage of vacuum cleaners: n° of hours/day years? <10□ 10-20□ 20-30 □ >30□ | | |
|  | Usage of dust masks: Frequent w/ filter □ Frequent w/o filter □ No/rare□ | | |
| Usage of products containing | Mercury□ Formaldehyde □ Arsenic □ Chlorophenols □ Solvents□ Other *(to be specified)* □ | | |

* Compile a job-specific card for every company of this sector given in full professional history

**Job-specific schedule**

1. METALLURGIC INDUSTRY

| Company* | | | Start | End |
| --- | --- | --- | --- | --- |
| Job sector/  Type of production | | Nickel refining (1) □ Stainless steel production (2) □ production of alloys or super-alloys (3) □Production of iron-chromium alloys (4) □ Productions of high-chromium content alloys. (5) □ | | |
| 1 | Production *(to be specified)* | N° hours/day how many years <5 □ 5-10 □ 10-20 □ 20-30□ >30 □ | | |
|  |  | Presence of exhaust systems? Yes localized □ Yes generalized □ No □ | | |
|  |  | Usage of dust masks: Frequent w/ filter□ Frequent w/o filter□ No/rare□ | | |
| 2 | Other production *(to be specified)* | N° hours/day how many years <5 □ 5-10 □ 10-20 □ 20-30□ >30 □ | | |
|  |  | Presence of exhaust systems? Yes localized □ Yes generalized □ No □ | | |
|  |  | Usage of dust masks: Frequent w/ filter□ Frequent w/o filter□ No/rare□ | | |
| 3 | Other production *(to be specified)* | N° hours/day how many years <5 □ 5-10 □ 10-20 □ 20-30□ >30 □ | | |
|  |  | Presence of exhaust systems? Yes localized □ Yes generalized □ No □ | | |
|  |  | Usage of dust masks: Frequent w/ filter□ Frequent w/o filter□ No/rare□ | | |
| 4 | Other production *(to be specified)* | N° hours/day how many years <5 □ 5-10 □ 10-20 □ 20-30□ >30 □ | | |
|  |  | Presence of exhaust systems? Yes localized □ Yes generalized □ No □ | | |
|  |  | Usage of dust masks: Frequent w/ filter□ Frequent w/o filter□ No/rare□ | | |
| 5 | Other production *(to be specified)* | N° hours/day how many years <5 □ 5-10 □ 10-20 □ 20-30□ >30 □ | | |
|  |  | Presence of exhaust systems? Yes localized □ Yes generalized □ No □ | | |
|  |  | Usage of dust masks: Frequent w/ filter□ Frequent w/o filter□ No/rare□ | | |
| 6 | Other production *(to be specified)* | N° hours/day how many years <5 □ 5-10 □ 10-20 □ 20-30□ >30 □ | | |
|  |  | Presence of exhaust systems? Yes localized □ Yes generalized □ No □ | | |
|  |  | Usage of dust masks: Frequent w/ filter□ Frequent w/o filter□ No/rare□ | | |
| 7 | Other production *(to be specified)* | N° hours/day how many years <5 □ 5-10 □ 10-20 □ 20-30□ >30 □ | | |
|  |  | Presence of exhaust systems? Yes localized □ Yes generalized □ No □ | | |
|  |  | Usage of dust masks: Frequent w/ filter□ Frequent w/o filter□ No/rare□ | | |
| 8 | Other production *(to be specified)* | N° hours/day how many years <5 □ 5-10 □ 10-20 □ 20-30□ >30 □ | | |
|  |  | Presence of exhaust systems? Yes localized □ Yes generalized □ No □ | | |
|  |  | Usage of dust masks: Frequent w/ filter□ Frequent w/o filter□ No/rare□ | | |
| Cleaning of workplace/ machineries | | Usage of compressed air: n° of hours/day years<10 □ 10-20 □ 20-30 □ >30 □ | | |
|  |  | Usage of vacuum cleaners: n° of hours/day years? <10□ 10-20□ 20-30 □>30□ | | |
|  |  | Usage of dust masks: Frequent w/ filter□ Frequent w/o filter□ No/rare□ | | |

* Compile a job-specific card for every company of this sector given in full professional history

**Job-specific schedule**

1. METAL TRANSFORMATION INDUSTRY

| Company* | | Start | End |
| --- | --- | --- | --- |
| Job sector/  Type of production |  | | |
| Welding occupation | N° hours/day how many years <5 □ 5-10 □ 10-20 □ 20-30□ >30 □ | | |
|  | Presence of exhaust systems? Yes localized □ Yes generalized □ No □ | | |
|  | Usage of dust masks: Frequent w/ filter□ Frequent w/o filter□ No/rare□ | | |
|  | Nickel □ Chromium □ Acids □ Solvents □ other (to be specified) □ | | |
| Galvanic processing | N° hours/day how many years <5 □ 5-10 □ 10-20 □ 20-30□ >30 □ | | |
|  | Presence of exhaust systems? Yes localized □ Yes generalized □ No □ | | |
|  | Usage of dust masks: Frequent w/ filter□ Frequent w/o filter□ No/rare□ | | |
|  | Nickel □ Chromium □ Acids □ Solvents □ other (to be specified) □ | | |
| Painting procedures | N° hours/day how many years <5 □ 5-10 □ 10-20 □ 20-30□ >30 □ | | |
|  | Presence of exhaust systems? Yes localized □ Yes generalized □ No □ | | |
|  | Usage of dust masks: Frequent w/ filter□ Frequent w/o filter□ No/rare□ | | |
|  | Type of paints used? Polyester p. □ Polyurethane p. □  Nitro p. □ Epoxy p. □ Other *(to be specified)* □ | | |
| Mechanical occupations | N° hours/day how many years <5 □ 5-10 □ 10-20 □ 20-30□ >30 □ | | |
|  | Presence of exhaust systems? Yes localized □ Yes generalized □ No □ | | |
|  | Usage of dust masks: Frequent w/ filter□ Frequent w/o filter□ No/rare□ | | |
|  | Nickel □ Chromium □ Acids □ Solvents □ other (to be specified) □ | | |
| Other production *(to be specified)* | N° hours/day how many years <5 □ 5-10 □ 10-20 □ 20-30□ >30 □ | | |
|  | Presence of exhaust systems? Yes localized □ Yes generalized □ No □ | | |
|  | Usage of dust masks: Frequent w/ filter□ Frequent w/o filter□ No/rare□ | | |
|  | Nickel □ Chromium □ Acids □ Solvents □ other (to be specified) □ | | |
| Cleaning of workplace/ machineries | Usage of compressed air: n° of hours/day years<10 □ 10-20 □ 20-30 □ >30 □ | | |
|  | Usage of vacuum cleaners: n° of hours/day years? <10□ 10-20□ 20-30 □ >30□ | | |
|  | Usage of dust masks: Frequent w/ filter□ Frequent w/o filter□ No/rare□ | | |

* Compile a job-specific card for every company of this sector given in full professional history

**Job-specific schedule**

1. CHEMICAL INDUSTRY (chromates production)

| Company* | | Start | End |
| --- | --- | --- | --- |
| Job sector/  Type of production |  | | |
| Production  *(to be specified)* | N° hours/day how many years <5 □ 5-10 □ 10-20 □ 20-30□ >30 □ | | |
|  | Presence of exhaust systems? Yes localized □ Yes generalized □ No □ | | |
|  | Usage of dust masks: Frequent w/ filter□ Frequent w/o filter□ No/rare□ | | |
| Other production *(to be specified)* | N° hours/day how many years <5 □ 5-10 □ 10-20 □ 20-30□ >30 □ | | |
|  | Presence of exhaust systems? Yes localized □ Yes generalized □ No □ | | |
|  | Usage of dust masks: Frequent w/ filter□ Frequent w/o filter□ No/rare□ | | |
| Cleaning of workplace/ machineries | Usage of compressed air: n° of hours/day years<10 □ 10-20 □ 20-30 □ >30 □ | | |
|  | Usage of vacuum cleaners: n° of hours/day years? <10□ 10-20□ 20-30 □>30□ | | |
|  | Usage of dust masks: Frequent w/ filter□ Frequent w/o filter□ No/rare□ | | |
| Usage of products containing | Nickel □ Chromium □ Arsenic □ Formaldehyde□ Other (to be specified)□ ………………………………………... | | |

* Compile a job-specific card for every company of this sector given in full professional history

**Job-specific schedule**

1. TEXTILE INDUSTRY

| Company* | | Start | End |
| --- | --- | --- | --- |
| Job sector/  Type of production | Cotton □ Wool □ Silk/ Synthetic fibers□ | | |
| Cotton carding | N° hours/day how many years <5 □ 5-10 □ 10-20 □ 20-30□ >30 □ | | |
|  | Presence of exhaust systems? Yes localized □ Yes generalized □ No □ | | |
|  | Usage of dust masks: Frequent w/ filter□ Frequent w/o filter□ No/rare□ | | |
| Wool carding | N° hours/day how many years <5 □ 5-10 □ 10-20 □ 20-30□ >30 □ | | |
|  | Presence of exhaust systems? Yes localized □ Yes generalized □ No □ | | |
|  | Usage of dust masks: Frequent w/ filter□ Frequent w/o filter□ No/rare□ | | |
| Silk/synthetic fiber carding | N° hours/day how many years <5 □ 5-10 □ 10-20 □ 20-30□ >30 □ | | |
|  | Presence of exhaust systems? Yes localized □ Yes generalized □ No □ | | |
|  | Usage of dust masks: Frequent w/ filter□ Frequent w/o filter□ No/rare□ | | |
| Filature/ weaving | N° hours/day how many years <5 □ 5-10 □ 10-20 □ 20-30□ >30 □ | | |
|  | Presence of exhaust systems? Yes localized □ Yes generalized □ No □ | | |
|  | Usage of dust masks: Frequent w/ filter□ Frequent w/o filter□ No/rare□ | | |
| Dyeing | N° hours/day how many years <5 □ 5-10 □ 10-20 □ 20-30□ >30 □ | | |
|  | Presence of exhaust systems? Yes localized □ Yes generalized □ No □ | | |
|  | Usage of dust masks: Frequent w/ filter□ Frequent w/o filter□ No/rare□ | | |
| Cleaning of workplace/ machineries | Usage of compressed air: n° of hours/day years<10 □ 10-20 □ 20-30 □ >30 □ | | |
|  | Usage of vacuum cleaners: n° of hours/day years? <10□ 10-20□ 20-30 □>30□ | | |
|  | Usage of dust masks: Frequent w/ filter□ Frequent w/o filter□ No/rare□ | | |

* Compile a job-specific card for every company of this sector given in full professional history

**Job-specific schedule**

1. FORESTRY

| Company* | | Start | End |
| --- | --- | --- | --- |
| Job sector/  Type of production |  | | |
| Wood hauling | N° hours/day how many years <5 □ 5-10 □ 10-20 □ 20-30□ >30 □ | | |
|  | Frequency: every day □ every week □ every month □ rarely□ | | |
|  | Usage of dust masks: Frequent w/ filter□ Frequent w/o filter□ No/rare□ | | |
| Undergrowth cleaning (using brush cutters) | N° hours/day how many years <5 □ 5-10 □ 10-20 □ 20-30□ >30 □ | | |
|  | Frequency: every day □ every week □ every month □ rarely □ | | |
|  | Usage of dust masks: Frequent w/ filter□ Frequent w/o filter□ No/rare□ | | |
| Other production *(to be specified)* | N° hours/day how many years <5 □ 5-10 □ 10-20 □ 20-30□ >30 □ | | |
|  | Frequency every day □ every week □ every month □ rarely □ | | |
|  | Usage of dust masks: Frequent w/ filter□ Frequent w/o filter□ No/rare□ | | |
| Other production *(to be specified)* | N° hours/day how many years <5 □ 5-10 □ 10-20 □ 20-30□ >30 □ | | |
|  | Frequency: everyday □ every week □ every month □ rarely □ | | |
|  | Usage of dust masks: Frequent w/ filter□ Frequent w/o filter□ No/rare□ | | |
| Exposure to any other chemical compounds? *(in particular to solvents)* Yes □ No □  *(to be specified)* | | | |

* Compile a job-specific card for every company of this sector given in full professional history

**Job-specific schedule**

9. OTHER (to be specified)

| Company* | | Start | End |
| --- | --- | --- | --- |
| Job sector/  Type of production |  | | |
| Production *(to be specified)* | N° hours/day how many years <5 □ 5-10 □ 10-20 □ 20-30□ >30 □ | | |
|  | Presence of exhaust systems? Yes localized □ Yes generalized □ No □ | | |
|  | Usage of dust masks: Frequent w/filter □ Frequent w/o filter □ No/rare □ | | |
| Other production *(to be specified)* | N° hours/day how many years <5 □ 5-10 □ 10-20 □ 20-30□ >30 □ | | |
|  | Presence of exhaust systems? Yes localized □ Yes generalized □ No □ | | |
|  | Usage of dust masks: Frequent w/filter □ Frequent w/o filter □ No/rare □ | | |
| Other production *(to be specified)* | N° hours/day how many years <5 □ 5-10 □ 10-20 □ 20-30□ >30 □ | | |
|  | Presence of exhaust systems? Yes localized □ Yes generalized □ No □ | | |
|  | Usage of dust masks: Frequent w/filter □ Frequent w/o filter □ No/rare □ | | |
| Other production *(to be specified)* | N° hours/day how many years <5 □ 5-10 □ 10-20 □ 20-30□ >30 □ | | |
|  | Presence of exhaust systems? Yes localized □ Yes generalized □ No □ | | |
|  | Usage of dust masks: Frequent w/filter □ Frequent w/o filter □ No/rare □ | | |
| Exposure to any other chemical compounds? *(in particular to solvents)* Yes □ No □  *(to be specified)* | | | |

* Compile a job-specific card for every company of this sector given in full professional history

| **Softwoods** | **Hardwoods** | **Exoticwoods** |
| --- | --- | --- |
| Fir | Maple | Afromosia |
| Cypress-Cedar | Alder | Ebony |
| Cypress | Birch | Iroko |
| Larch | American walnut | Balsa wood |
| Spruce | White beech | Kauriwood |
| Pine tree | Ash | Limba wood |
| Douglas fir | Chestnutwood | Merantiwood |
| Giant sequoia | Beech | AfricanMahogany |
| Cupressaceae | Walnutwood | Mansoniawood |
| Pinaceae | American sycamore | Obeche |
|  | Poplar | Rosewood |
|  | Cherry wood | Brazilianrosewood |
|  | Oak | Rimu |
|  | Willow | Teak |
|  | Linden |  |
|  | Elm |  |
|  | Oak |  |
